# Flexible Organic Field‐Effect Transistor (OFET) Based 2T0C DRAM Cells with 2‐Bit Operation and Extended Retention

**DOI:** 10.1002/advs.202500300

**Published:** 2025-03-07

**Authors:** Xuemeng Hu, Zhenhai Li, Tianyang Feng, Jialin Meng, Qingxuan Li, Hao Zhu, Qingqing Sun, David Wei Zhang, Lin Chen

**Affiliations:** ^1^ School of Microelectronics State Key Laboratory of Integrated Chip and System Fudan University Shanghai 200433 P. R. China; ^2^ School of Integrated Circuits Shandong University Jinan 250100 China; ^3^ School of Integrated Circuits Anhui University Anhui 230601 P. R. China; ^4^ National Integrated Circuit Innovation Center Shanghai 201201 China

**Keywords:** finite element analysis, flexible 2T0C DRAM cells, long retention, multilevel storage, organic field effect transistors

## Abstract

The emerging demand of data storage in wearable devices, flexible circuits based on organic semiconductor materials are encouraged, while organic field effect transistors face the challenges of low operating voltage and high on/off ratio. In this work, a high‐performance flexible organic field effect transistor (OFET) is built with a threshold voltage range of −0.45 to −0.86 V and an on/off ratio between 10^6^ and 10^8^. The subthreshold swing of the OFETs is lower than 60 mV dec^−1^. Through finite element analysis, the OFET shows excellent mechanical stability owing to the stress relief during the bending process. Furthermore, a flexible 2T0C DRAM cell is demonstrated based on the OFETs. The stable electrical characteristics of the OFETs enable the 2T0C DRAM cell to achieve a retention time exceeding 300 s under both initial and bending conditions. Additionally, 2‐bit memory operations are realized by adjusting the voltage of the word bit line (*V*_WBL_) and the voltage of the word write line (*V*_WWL_), demonstrating consistent performance in both the initial and after‐bending state.

## Graphical abstract

A flexible 2T0C DRAM cells which consist of two high‐performance organic field‐effect transistors is reported, which exhibiting exceptional flexibility and multilevel storage characteristics. That device achieves a remarkable retention time exceeding 300 s, both in its initial state and under bending conditions, making it a promising candidate for future flexible memory electronic applications.

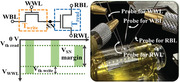

## 1 Introduction

Organic field‐effect transistors (OFETs) have garnered significant attention due to their compatibility with large‐area, cost‐effective fabrication methods such as spin coating and vacuum thermal evaporation.^[^ ^1^, ^2^, ^3^, ^4^ ^]^ These fabrication techniques are not only scalable but also allow for the production of high‐performance devices at relatively low costs, making them ideal for industrial applications.^[^ ^5^, ^6^ ^]^ In recent years, the unique mechanical properties of organic semiconductors, particularly their low Young's modulus, have positioned OFETs as a promising technology for the development of flexible and stretchable electronics.^[^ ^7^, ^8^, ^9^ ^]^ This intrinsic flexibility enables OFETs to conform to non‐planar surfaces without compromising their performance, paving the way for their integration into a wide range of innovative applications such as electronic‐paper displays, microprocessors, flexible sensors, electronic skins, and wearable integrated circuits.^[^ ^10^, ^11^, ^12^, ^13^ ^]^

Dynamic random‐access memory (DRAM) is a crucial element in modern computing systems, serving as the primary memory storage in a wide range of digital devices, from smartphones and personal computers to servers and embedded systems. It plays an indispensable role in the advancement of several key technologies, including cloud computing, edge computing, the internet of things, and Artificial Intelligence (AI).^[^ ^14^, ^15^, ^16^ ^]^ These technologies rely heavily on large‐scale data processing and real‐time responsiveness, where DRAM provides fast, volatile storage to support the high‐speed operation of processors.^[^ ^17^, ^18^ ^]^ However, despite its widespread use, contemporary DRAM technology faces several significant challenges that impact both its performance and power consumption.^[^ ^19^, ^20^ ^]^ These challenges are collectively referred to as the “memory wall,” which refers to the increasing disparity between the speed of processors and the speed of memory access. As processor speeds have advanced, the limitations of DRAM in terms of access time and bandwidth have become more apparent.^[^ ^19^, ^21^ ^]^ Addressing these issues is critical for the future scalability and performance of DRAM in increasingly demanding computational environments. To overcome these limitations and achieve a new generation of DRAM technology, it is essential to design transistors and circuits with low driving voltages, minimal static and dynamic power consumption, high gain, and rapid response times.^[^ ^22^ ^]^

In this work, a high‐performance C8‐BTBT OFET was fabricated on the flexible muscovite substrate. The fabricated OFET exhibits an on/off ratio between 10^6^ and 10^8^ and transistors show the minimum SS of 17 mV dec^−1^ at a drain current of 10^−12^ A. Besides, the threshold voltage ranges from −0.45 to −0.86 V, exhibiting decent uniformity. The thermal budget of the entire process remains below 300 °C, ensuring compatibility with backend‐of‐the‐line (BEOL). After multiple repeated bending, the electrical characteristic of the OFETs has no significant change. Meanwhile, finite element analysis (FEA) was conducted by ABAQUS of the OFET to simulate the distribution of stress across the interfaces and the bulk for each layer of the OFET. The OFETs serve as write and read transistors for flexible 2T0C DRAM cell, which has a retention time exceeding 350 s and can store 2 bits of data. Furthermore, after multiple bending cycles, the retention time does not significantly decrease, and the cell remains to achieve the polymorphic storage functionality, demonstrating excellent flexibility. The 2T0C DRAM cell exhibits significant potential for future wearable circuit development.

## 2 Experimental Section

**Figure 1:**
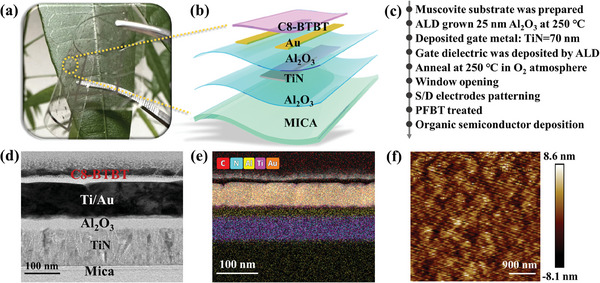
a) Photograph of the flexible organic field‐effect transistor in a bent state b) Schematic diagram of the flexible OFET. c) The critical experimental method flow of the flexible OFET fabrication process. d) HRTEM image of the C8‐BTBT/Ti/Au/Al_2_O_3_/TiN/Mica stack. e) The EDX mapping images of C8‐BTBT OFETs structure. f) Atomic force microscopy (AFM) AFM analysis of the surface of the OFETs.

The optical image of a flexible OFET prepared on a mica substrate in the bent state is shown in **Figure** **1**a. Meanwhile, Figure 1b shows an enlarged schematic diagram of a single OFET. TIN and Ti/Au were used as the bottom gate and source/drain (S/D) electrodes of the transistor, respectively. The OFET adopts a bottom gate bottom contact structure, and the fabrication process is shown in Figure 1c.

Flexible muscovite (mica) substrate was first peeled from a mica flake and cleaned with acetone, isopropyl alcohol, and deionized water, and then dried in nitrogen. Then a 10 nm Al_2_O_3_ was deposited on the mica substrate to improve the interface of the substrate and device. The bottom gate of C8‐BTBT OFETs was patterned by UV lithography, then an 80 nm TiN metal electrode was deposited by physical vapor deposition (PVD). Al_2_O_3_ (25 nm) deposited using atomic layer deposition at 250 °C as gate dielectric. The OFETs subsequently anneal at 250 °C for one hour in an oxygen atmosphere to reduce oxygen vacancy defects in the dielectric. After annealing the interconnect window was opened using reactive ion etching. S/D interdigitated structure electrodes were also patterned by UV lithography and Ti/Au were deposited by PVD. The thickness of the deposited Ti and Au electrodes were 10 and 70 nm, respectively. Ti was used as an adhesive layer to ensure that Au electrodes would not fall off in subsequent processes. In the coplanar bottom‐contact configuration of OFETs, the interface between metal and organic materials exhibited critical parameters that differed significantly from those of thin‐film transistors.^[^ ^23^ ^]^ OFETs were generally fabricated using organic semiconductors as the channel material, which were characterized by a low density of thermal carriers.^[^ ^4^, ^24^, ^25^ ^]^ Therefore, in the absence of a highly doped contact region, the electrostatic influence of the built‐in potential at the contact did not diminish into a depletion region but rather diffuses throughout the bulk of the organic semiconductor.^[^ ^26^ ^]^ Furthermore, Fermi‐level pinning created a macroscopic bottleneck for current flow due to the charge injection barrier, manifesting as significant contact resistance (*R*_c_).^[^ ^27^ ^]^ Extensive studies had demonstrated that thiolate self‐assembled monolayers (SAMs) on Au could effectively optimize the interface in OFETs. In this work, PFBT SAMs were used to reduce *R*_c_.^[^ ^26^, ^28^ ^]^ Finally, organic semiconductor C8‐BTBT was deposited onto the surface of the S/D electrodes by vacuum thermal evaporation. The thermal budget of the entire process remained below 300 °C, ensuring compatibility with BEOL.

## 3 Results and Discussion

The high‐angle annular dark‐field scanning transmission electron microscopy has shown in Figure 1d, which clearly shows the structure of C8‐BTBT OFETs. Figure 1e is the corresponding energy dispersive X‐ray (EDX) elemental mapping images of the OFET. The EDX mapping image of each element in the OFET is shown in Figure S1 (Supporting Information). Atomic force microscopy (AFM) was used to analyze the surface morphology of the C8‐BTBT organic film. As shown in Figure 1f the surface of the organic film of the OFET is relatively flat, and it can be seen from Figure S2a (Supporting Information) that the root‐mean‐square roughness is 1.8 nm. The high‐quality C8‐BTBT film ensures the performance of the device.

**Figure 2:**
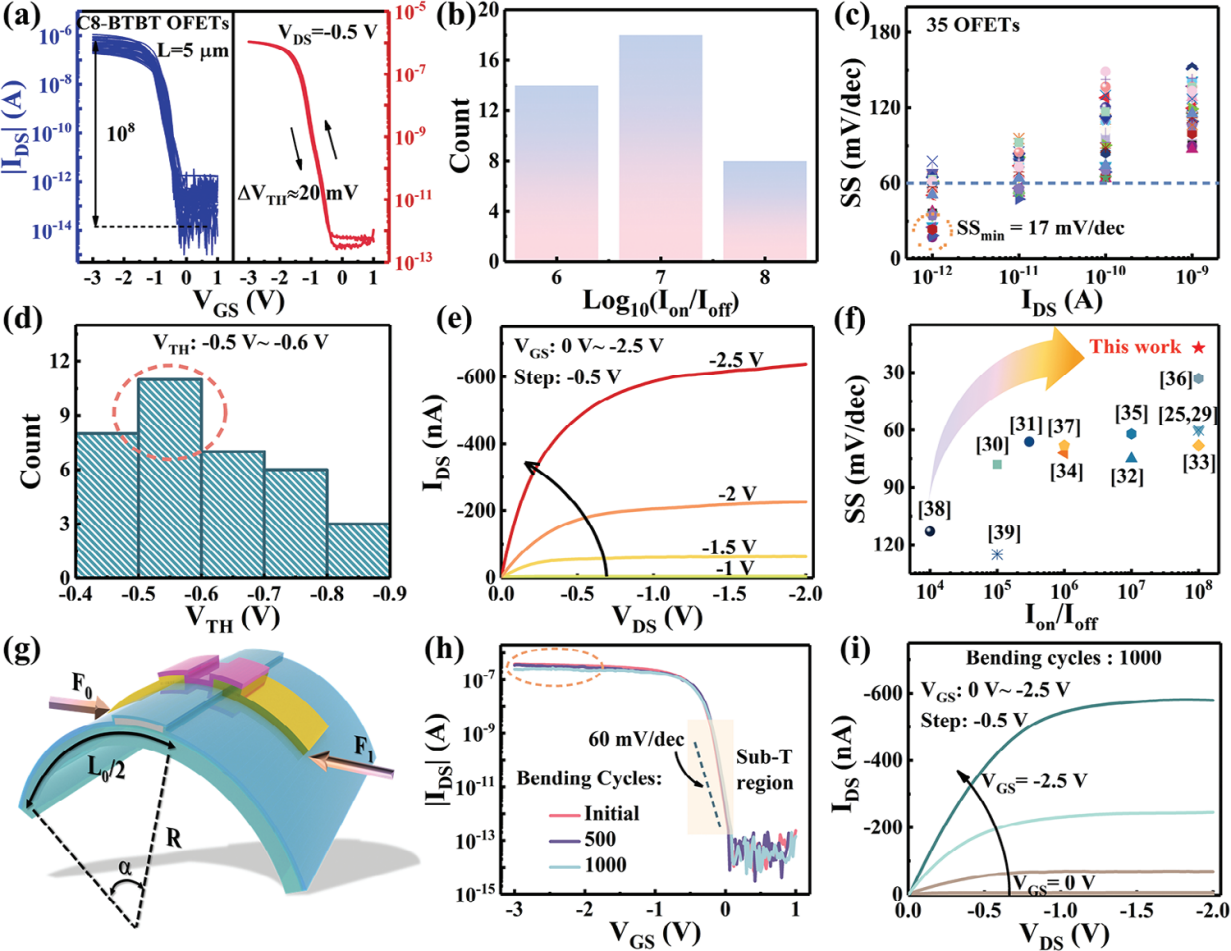
a)Transfer characteristics of 35 different C8‐BTBT transistors, the transistor has a small *V*_th_ offset under *V*_GS_ dual‐sweeping state. b) Histogram of ON/OFF ratio (*I*_on_/*I*_off_) from 35 C8‐BTBT OFETs, exhibiting decent uniformity. c) The SS distribution of the OFETs, some transistors have SS below 60 mV dec^−1^ in the subthreshold region, showing a minimum SS of 17 mV dec^−1^. d) Histogram of the threshold voltage of the OFETs, the distribution of the *V*_th_ indicating the OFETs has excellent uniformity. e) Output characteristic of the C8‐BTBT OFET. f) The benchmark for comparing the performance of the OFETs with recent references on transistors. g) Schematic of the C8‐BTBT OFETs under a bending diameter of 18 mm. h) Transfer characteristics for the OFETs after different bending cycles with a bending diameter of 18 mm. i) Output characteristic of the OFETs after 1000 bending cycles with a bending diameter of 18 mm.

**Figure** **2**a presents the transfer characteristics of 35 different C8‐BTBT OFETs with channel lengths of 5 mm. The threshold voltage exhibits a small shift of ≈20 mV shift during the gate voltage dual‐sweeping process. After annealing in an oxygen atmosphere, the hysteresis of OFETs during the gate voltage dual sweeping process was significantly improved, and the transfer characteristics changed at different annealing hours as shown in Figure S3 (Supporting Information). An oxygen atmosphere will fill the oxygen vacancies on the interface of the Al_2_O_3_ dielectric and C8‐BTBT channel at high temperatures, thereby improving the hysteresis of transistor transfer characteristics.^[^ ^29^ ^]^ The comparison for the transfer characteristics of OFETs with and without PFBT SAMs treated Au electrodes is shown in Figure S4b (Supporting Information). The formation of the PFBT SAM significantly increases the drain current in the saturation region after the transistor turns on, and it renders the transfer characteristics curve steeper, thereby enhancing the overall performance of the transistor. The ratio of the “on” state drain current to the “off” state current for the OFETs is ≈10^8^, with the statistics histogram of the on/off ratio of transfer characteristics is shown in Figure 2b. The on/off ratio for the counted 35 OFETs are greater than 10^6^, and some organic transistors reaching an on/off ratio as high as 10^8^. The subthreshold swing (SS) of these OFETs at different ranges of drain current is shown in Figure 2c. When the drain current is in the range of 10^−12^–10^−11^ A, the transistors are in the sub‐threshold region. For some devices, the SS was found to be less than 60 mV dec^−1^, with the lowest subthreshold swing reaching 17 mV dec^−1^ at the drain current is 10^−12 ^A. Histogram of subthreshold swing from 35 C8‐BTBT OFETs has shown in Figure S5 (Supporting Information). Figure 2d shows the distribution of threshold voltage (*V*_th_) for the C8‐BTBT OFETs, where the majority of the transistors have a threshold voltage between −0.4 and −0.6 V. The range of *V*_th_ was from −0.45 to −0.86 V, with an average value of −0.6 V. The ″box‐and‐whisker plot of *V*_th_ for the OFETs is shown in Figure S4a (Supporting Information), further revealing decent uniformity. The threshold voltage is estimated by analyzing the subthreshold region of the transfer characteristics for the OFETs and using linear extrapolation fitting, as shown in Figure S4b (Supporting Information).^[^ ^30^ ^]^ Figure 2e shows the output characteristic of OFETs, V_GS_ of the transistor ranges from 0 to −2.5 V, the step of *V*_GS_ is −0.5 V. When *V*_GS_ is less than −1 V, the transistor is in the off state. The transistor gradually turns on and enters the linear and saturation regions as *V*_GS_ and *V*_DS_ increase. Figure 2f benchmarks the *I*_on_/*I*_off_ and SS performance of the OFETs reported in this work (shown as a red five‐point star) against those references about OFET and oxide semiconductor transistors (shown as other symbols).^[^ ^25^, ^30^, ^31^, ^32^, ^33^, ^34^, ^35^, ^36^, ^37^, ^38^, ^39^, ^40^ ^]^ It can be found that the OFET proposed in this work has excellent performance in terms of *I*_on_/*I*_off_ and SS.

The OFET that fabricated on a flexible Mica substrate, further investigation about its bending characteristics was conducted. Figure 2g illustrates the schematic diagram of the bending strain induced by applying stress to both sides while keeping the center point of the device fixed. The comparison for the transfer characteristics of the OFET between after multiple bending with a bending diameter of 18 mm and the initial state is shown in Figure 2h. It can be seen that the device retains steep transfer characteristic curves with SS below 60 mV dec^−1^ after multiple bending cycles. However, the drain current decreases after multiple bends at higher *V*_GS_, which may be related to an increase in gate leakage current in the transistor (Figure S6, Supporting Information). This could be due to the difference in Young's modulus between the dielectric layers and electrode, such as TiN and Al_2_O_3_, in the OFETs, leading to cracks at the edges, as shown in Figure S7 (Supporting Information). Figure 2i shows the output characteristics of the device after 1000 bending cycles, revealing no significant changes compared to Figure 2e, which demonstrates that the OFET can maintain relatively stable electrical performance after repeated bending.

**Figure 3:**
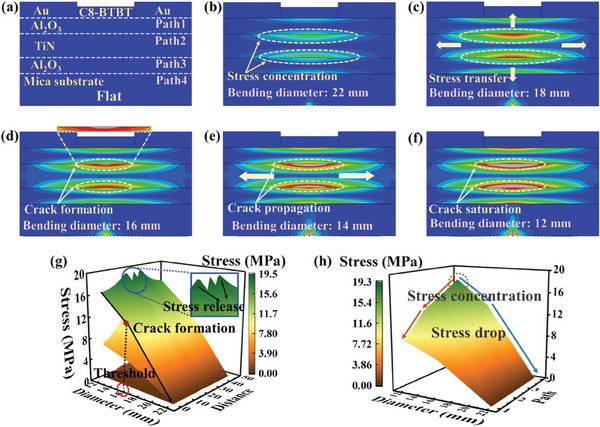
Stress variation of the OFET during bending analyzed by FEA. a–f) The formation and propagation of stress and cracks in each layer of the OFET based on different bending diameters. g) Stress as a function of the bending diameter of the OFET. h) Stress variation with different diameters in different paths.

In order to investigate the cause of electrical performance degradation after repeated bending of the OFETs, finite element analysis (FEA) was conducted by ABAQUS to model the OFET and study the stress distribution of the OFET, as shown in **Figure** **3**. Figure 3a–f illustrates the 2D model of the transistor and the gradual change in internal stress as the OFET undergoes bending, with the bending state from flat to the diameter reduced to 12 mm. The different bending diameters are achieved by varying the force applied at both ends of the OFET. The mesh of the 2D model is shown in Figure S7 (Supporting Information). Four paths were set at the interfaces of each layer of the device during stress simulation, as shown in Figure 3a. When the bending diameter is 22 mm, stress occurs in both the Al_2_O_3_ and TiN layers. It can be seen from Figure 3b, the stress is mainly concentrated in the center of the Al_2_O_3_ – TiN interface. As the applied force increased (i.e., as the bending diameter decreased), the stress transfers from the center of the interface, and the stress at the center further increases, as shown in Figure 3c. When the bending diameter is reduced to 16 mm, cracks begin to form at the Al_2_O_3_–TiN–Al_2_O_3_ interface. As the bending diameter continues to decrease, cracks in the bulk of the TiN layer enlarge, propagating from the center toward the sides, ultimately resulting in failure in the regions surrounding the cracks. With further reduction in the bending diameters, the cracks in the bulk of the TiN layer reach saturation when the bending diameter is at 12 mm. The simulation results illustrating the progression of cracks from initiation to saturation are presented in Figure 3d–f. Figure 3g,h shows the stress analysis at different distances and paths under various bending diameters.

Figure 3g shows that when the bending diameter is reduced to less than 18 mm, the stress on the OFETs increases gradually, indicating that 18 mm is the threshold for device bending. When the bending radius is smaller than 18 mm, stress in the bulk of each layer may lead to OFET failure. The enlarged image in Figure 3g demonstrates the stress distribution at the central of the Al_2_O_3_–TiN–Al_2_O_3_ interfaces. Stress is released at the interfaces between each two layers, reduce the overall stress of OFET, decrease the possibility of crack formation at other locations, and improve the bending durability and flexibility of OFET. Therefore, the Al_2_O_3_ deposited on the mica substrate not only improves the interface but also enhances the flexibility of the device. Figure S8 (Supporting Information) shows the stress distribution of each path. Figure 3h demonstrates that the stress is primarily concentrated in path 4 at smaller bending diameters, indicating that the formation of cracks relieves the stress at the Al_2_O_3_—TiN–Al_2_O_3_ interfaces, allowing the stress to propagate downward. This observation explains the reduction in drain current when the OFET is bent and establishes a theoretical foundation for the further development of flexible OFETs.

**Figure 4:**
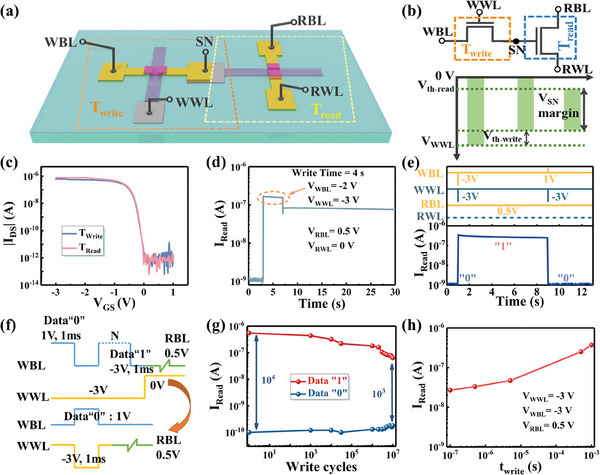
a) The schematic diagram of the 2T0C DRAM cell based on the high‐performance flexible OFETs. b) Circuit schematic and store node margin of the 2T0C DRAM cell. c) Transfer characteristic of write and read transistors of the 2T0C DRAM cell. The lower *V*_th_ of the transistors ensures the store node has a higher effective sensing window. d) The relationship between read current and time of the 2T0C DRAM cell. e) The operation of writing data “1” and data “0” into the 2T0C DRAM cell within a 10 s period. f) Pulse sequence for endurance test. g) Performance of the endurance for the 2T0C cell. High endurance exceeding 10^7^. h) Read the current of the 2T0C DRAM cell with different write times.

The schematic diagram of the 2T0C DRAM cell based on the high‐performance flexible OFETs is shown in **Figure** **4**a. The 2T0C DRAM cell consists of two OFETs, where the charge is stored in the gate capacitance of the read transistor (*T*_read_). The write transistor (*T*_write_) controls the charging and discharging of the *T*_read_ gate capacitance, which can be monitored by the output current of the *T*_read_. Figure 4b presents the circuit schematic of the 2T0C DRAM cell and the voltage margin that sensing for the storage node (SN). Charges can be stored at the storage node and monitored by the output current of the read transistor when both the write transistor and the read transistor are turned on. Therefore, the storage node voltage (*V*_SN_) should be lower than the write word line voltage (*V*_WWL_) by the *V*_th_ of the write transistor, while it also should be higher than the *V*_th_ of the read transistor. The optical image of the 2T0C DRAM cell is shown in Figure S10a (Supporting Information). Figure 4c shows the transfer characteristics of the two OFETs that form the 2T0C DRAM cell, with a *V*_th_ of ≈−0.5 V. The small *V*_th_ ensures that the storage node can obtain a larger voltage margin.

**Figure 5:**
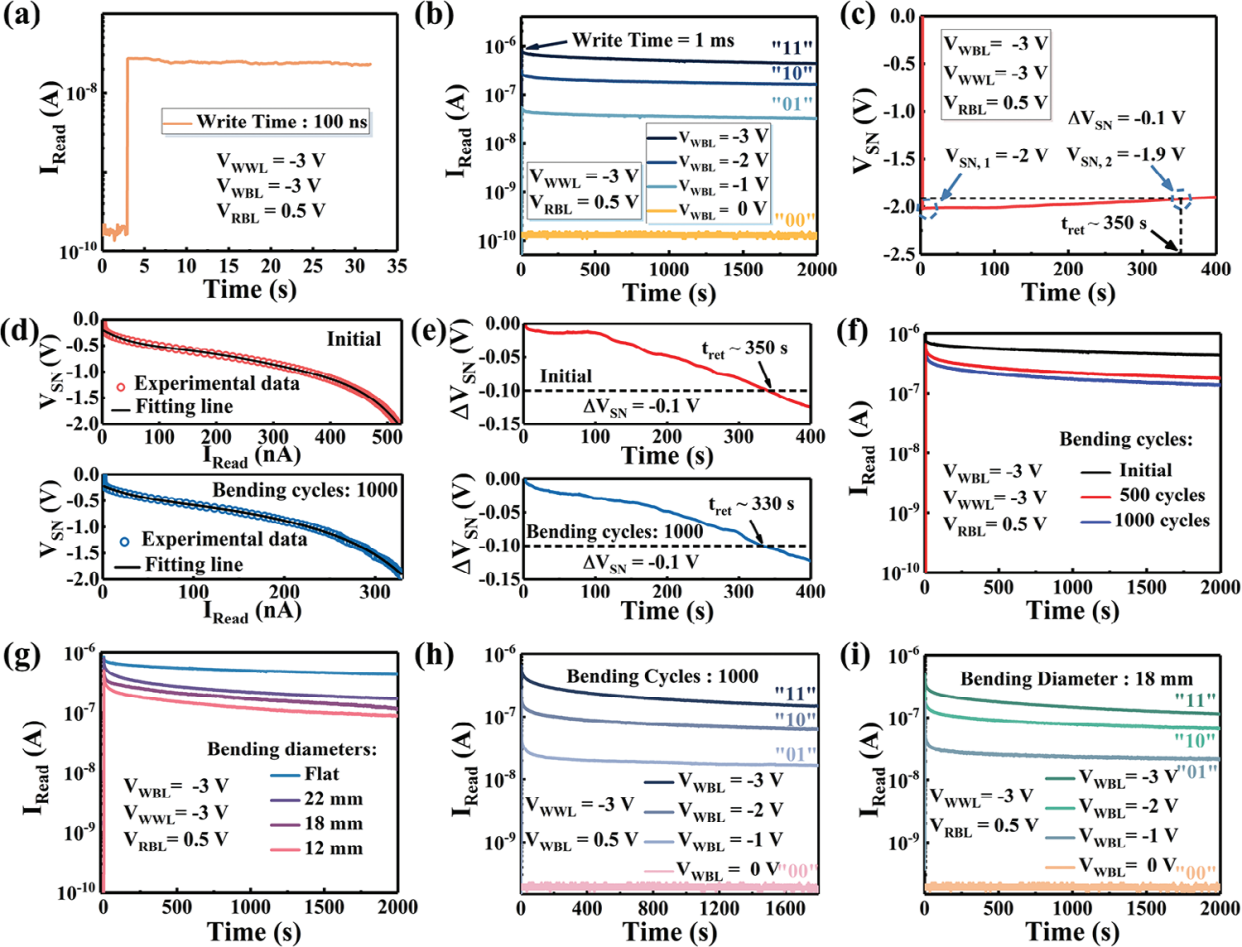
a) The relationship between the *I*_Read_ of the 2T0C DRAM cell and time at a write time of 100 ns. b) Read current of the 2T0C DRAM cell with different *V*_WBL_. 2bit data retention is obtained. c) *V*_SN_ as a function of retention time for the 2T0C DRAM cell. Using the 0.1 V‐drop norm, 350 s retention time is obtained. d) Polynomial fitting of *V*_SN_–*I*_Read_ data for initial and after 1000 bending cycles. e) Comparison of ∆*V*_SN_ over time of 2T0C DRAM cell for initial and after 1000 bending cycles. f,g) are the retention characteristics of the 2T0C cell for different bending cycles and bending diameters, respectively. h) After 1000 bending cycles the 2T0C DRAM cell can maintain 2‐bit data retention. i) Under the bending diameter of 18 mm, the 2T0C DRAM cell can realize 2‐bit multilevel data storage.

In order to investigate the memory performance of the 2T0C DRAM cell, a voltage of −2 and −3 V is applied to the write bit line (WBL) and write word line (WWL), respectively, with a write time of 4 s, the function of the read current (*I*_Read_) and time is shown in Figure 4d. The voltage on the read bit line (RBL) of the read transistor is held at 0.5 V to maintain the read current state. It is observed that after the *V*_WWL_ and *V*_WBL_ are removed, the 2T0C DRAM cell maintains a high *I*_Read_, indicating that charge is effectively stored in the gate capacitance of the read transistor. Figure 4e illustrates the operation of writing data “1” and data “0” into the 2T0C DRAM cell within a 10 s period. The *V*_WWL_ is −3 V which ensures the data can be stored in the 2T0C DRAM cell, while the *V*_WBL_ is −3 and 1 V to write the data “1” and data “0”, respectively. The endurance of the 2T0C DRAM cell is measured by repeatedly writing data “1” and data “0” operations using the pulse sequence shown in Figure 4f, with the results shown in Figure 4g. In one period, the *V*_WBL_ is a −3 and 1 V square wave to continuously write data “1” and data “0”. The *V*_WBL_ ends at −3 V for writing data “1”. During that process, the *V*_WWL_ is −3 V to ensure the 2T0C DRAM cell can store data, while the *V*_RBL_ is 0.5 V, maintaining the 2T0C DRAM cell in the read state. The variation of the *I*_Read_ during this process is shown in Figure S10b (Supporting Information). Afterward, the *V*_WBL_ is 1 V to write data “0”. It can be seen from Figure 4g, that the 2T0C DRAM cell initially has a storage window of 10^4^, and it still maintains a 10^3^ storage window after 10^7^ program‐erase cycles, demonstrating excellent endurance of the 2T0C DRAM cell. Figure 4h shows the relationship between *I*_Read_ and write time of the 2T0C DRAM cell. As the write time decreases, the *I*_Read_ also decreases. When the write time is 100 ns, the *I*_Read_ of the 2T0C DRAM cell is 27 nA, indicating that the memory cell has a fast response speed. The relationship between the *I*_Read_ of the 2T0C DRAM cell and time at a write time of 100 ns is shown in **Figure** **5**a.

Figure 5b,c exhibit the relationship between *I*_Read_ and time, as well as the relationship between storage node voltage and time, respectively. The voltage of the storage node can be extracted from the previously measured transfer characteristics of the read transistor and the *I*_Read_. As shown in Figure 5b, by varying the *V*_WBL_, the *I*_Read_ of the 2T0C DRAM cell can be distinctly differentiated, demonstrating the 2T0C DRAM cell can realize 2‐bit multilevel data storage characteristics.^[^ ^21^, ^41^ ^]^ The write time is 1 ms, with the *V*_WWL_ and *V*_RBL_ is −3 and 0.5 V, respectively. The 2T0C DRAM cell exhibits excellent retention of *I*_Read_ over a testing period of 2000 s. The extended retention time for *I*_Read_ is shown in Figure S11a (Supporting Information), where the I_Read_ persists for up to 6000 s or even longer after writing data “1.” In Figure 5c, when the *V*_WBL_ is −3 V to write data “1,” the VSN jumps from 0 to −2 V, and then gradually decreases. After ≈350 s, the storage node voltage drops to −1.9 V. Using a voltage drop of 0.1 V as a benchmark for retention failure, our 2T0C DRAM cell realizes a long retention time of 350 s.^[^ ^42^ ^]^

In addition, the flexible characteristics of the 2T0C DRAM cell were also tested. Figure 5d shows the relationship between the *V*_SN_ and the *I*_Read_ before and after 1000 bending cycles. The bending diameter is 18 mm. The relationship between the *V*_SN_ and time before and after bending is shown in Figure 5e. The storage node voltage can be extracted from the relationship between the *V*_SN_ and the *I*_Read_ of the read transistors.^[^ ^21^, ^41^ ^]^ After 1000 bending cycles, the retention time of the 2T0C DRAM cell decreases from 350 to 330 s. This decrease is likely due to the cracks that develop in the write and read transistors after multiple bending cycles, which affect the gate capacitance of the read transistor, leading to the degradation of retention time. During the bending test, fix the 2T0C DRAM cell onto a small bottle of a certain diameter using high‐temperature tape, as shown in Figure S11b (Supporting Information).

Figure 5f–i show the relationship between the read current and time, under different bending cycles and bending diameters. Figure 5f,g illustrates the variation in the read current of the 2T0C DRAM cell after writing data “1,” under different bending cycles and bending diameters. It can be observed that, although the read current exhibits a degraded trend compared to the initial state after multiple bending cycles or under different bending diameters, the memory cell still maintains long‐term retention of the read current. The degradation may be caused by an increase in the internal stress of the memory cells as the bending diameter decreases, which corresponds to the results of finite element analysis, leading to an increase in the leakage current of OFETs, as shown in Figure S4 (Supporting Information). The increased leakage current affects the stability of the storage node voltage, which in turn causes a reduction in the read current. Furthermore, repeated bending may cause material fatigue or microstructural degradation, such as crack formation (Figure S7, Supporting Information). Figure 5h,i demonstrate that whether after repeated bending cycles or at different bending diameters, applying different *V*_WBL_ allows the 2T0C DRAM to realize 2‐bit multilevel data storage characteristics. Table S1 (Supporting Information) shows the performance comparison of the flexible 2T0C DRAM cells and recent studies. Compared to recent research, the 2T0C DRAM cells achieve retention comparable to that of oxide semiconductor‐based 2T0C DRAM cells under the same retention standards, while also offering multilevel storage characteristics. Besides, the 2T0C DRAM cell also has excellent mechanical flexibility. This confirms that our flexible 2T0C DRAM cell exhibits excellent flexibility, maintaining stable storage performance even after repeated bending and under various bending diameters. That paves the way for the development of flexible and wearable integrated circuits, laying a solid foundation for future advancements in flexible wearable technology.

## 4 Conclusion

In summary, we successfully fabricated high‐performance and stable C8‐BTBT OFET on a flexible mica substrate, with a *V*_th_ range from −0.45 to −0.86 V and an on/off ratio of 10^8^. The SS of the flexible OFET is lower than 60 mV dec^−1^. The entire fabrication process for the OFETs remains below 300 °C, ensuring compatibility with BEOL. The stress distribution of the OFETs under the bending state was conducted using finite element analysis. The cracks formed after bending can effectively release stress and enhance the flexibility endurance of the OFET, leading to the excellent flexibility of OFET. Furthermore, the OFETs was served as write and read transistors for high‐performance flexible 2T0C DRAM cells. This flexible 2T0C DRAM cell has a retention time of over 350 s and an endurance over 10^7^ cycles. Meanwhile, a 2‐bit multilevel data storage characteristic of the 2T0C DRAM cell is realized and maintains stable performance after multiple bends. The retention time can remain stable compared to the initial state and it can still obtain multilevel data retention. This work demonstrates the 2T0C DRAM cell has excellent data retention, mechanical flexibility, and reliable operation under bending conditions, exhibiting significant potential for future wearable circuit development.

## Conflict of Interest

The authors declare no conflict of interest.

## Supporting information



Supporting Information

## Data Availability

The data that support the findings of this study are available from the corresponding author upon reasonable request.
