# Supplementary material for: Flexible Organic Field‐Effect Transistor (OFET) Based 2T0C DRAM Cells with 2‐Bit Operation and Extended Retention
Source: Adv Sci (Weinh). 2025 Mar 7;12(17):2500300. doi: 10.1002/advs.202500300 (PMC12061271; doi:10.1002/advs.202500300)
Supplement: Supplementary file 1 — Supporting Information [file ADVS-12-2500300-s001.docx]

**Flexible Organic Field-Effect Transistor (OFET) Based 2T0C DRAM Cells with 2-bit Operation and Extended Retention**

Xuemeng Hu^1^, Zhenhai Li^3^, Tianyang Feng^1^, Jialin Meng^2,4*^, Qingxuan Li^3*^, Hao Zhu^1,4^, Qingqing Sun^1,4^, David Wei Zhang^1,4^, and Lin Chen^1,4*^

^1^School of Microelectronics, State Key Laboratory of Integrated Chip and System, Fudan University, Shanghai 200433, P. R. China,

^2^School of Integrated Circuits, Shandong University, Jinan 250100, China

^3^School of Integrated Circuits, Anhui University, Anhui 230601, P. R. China

^4^National Integrated Circuit Innovation Center, Shanghai 201203, China

**Email: jlmeng@sdu.edu.cn; liqx@ahu.edu.cn; linchen@fudan.edu.cn*

**The supporting information includes:**

**Fig. S1** EDS mapping images of the C8-BTBT organic field effect transistors

**Fig. S2** (a) Atomic force microscope (AFM) image of the surface morphology of the OFET (b) The comparison of the transfer characteristic of the OFET with and without treated by PFBT before source and drain electrodes was deposited.

**Fig. S3** The variation of OFETs transfer characteristic curve under different annealing times.

**Fig. S4** (a) The “box-and-whisker plot of Vth for the 35 different OFETs. (b) The transfer characteristics for the OFETs to estimate the Vth of the OFETs.

**Fig. S5** Histogram of Subthreshold Swing (SS) Statistics for 35 OFETs.

**Fig. S6** (a) and (b) are the leakage current of the OFETs under different bending cycles and bending diameters.

**Fig. S7** Schematic diagram of changes in the dielectric layer of the OFETs under bending state.

**Fig. S8** The finite element analysis model for the OFETs.

**Fig. S9** Stress as a function of bending diameter at the (a) path 1, (b) path 2, (c) path 3, (d) path 4.

**Fig. S10** (a) The optical image of the 2T0C DRAM cell constructed by the high performance OFETs. (b) The variation of the IRead during the endurance test process.

**Fig. S11** (a) The relationship of the IRead and time over 6000 s. (b) Real-time test photos of the 2T0C DRAM cell.

**Table 1** Performance comparison of our 2T0C DRAM cells and recent studies.

**S1 EDS mapping images of the C8-BTBT organic field effect transistors**


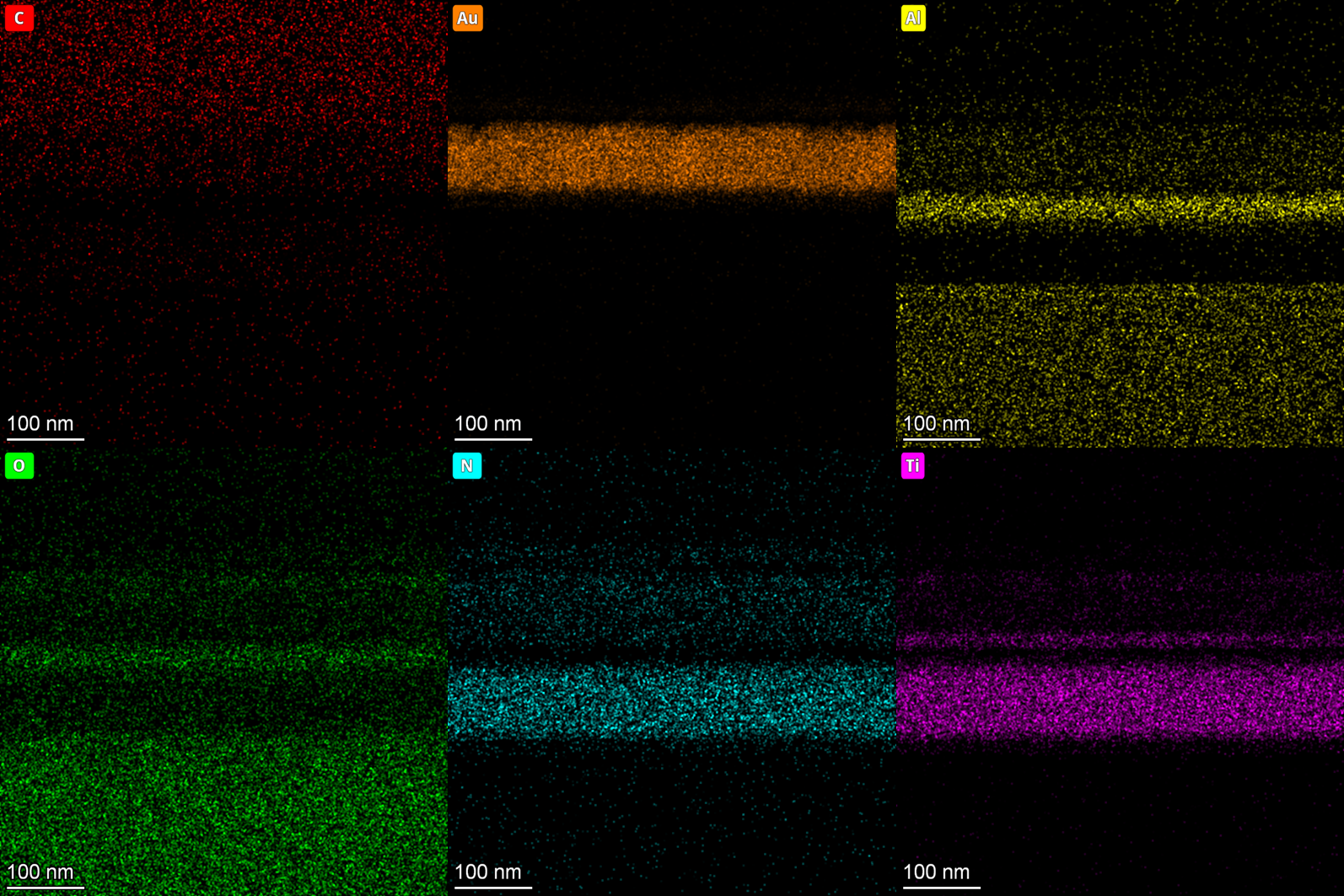


**Fig. S1** EDS mapping image of the C8-BTBT/Au/Al_2_O_3_/TiN/Al_2_O_3_/Mica

Fig. S1 the cross sectional image of the C8-BTBT/Au/Al_2_O_3_/TiN/Al_2_O_3_/Mica OFETs is clear. In order to obtain the element distribution, energy dispersive spectrometer mapping images are used as the measurement method. The constituent elements of C, O, Ti, N, Au and Al are uniformly distributed in the films.

**S2** **(a) Atomic force microscope (AFM) image of the surface morphology of C8-BTBT (b) The transfer characteristic of the OFET with and without treated by PFBT before source and drain electrodes was deposited.**


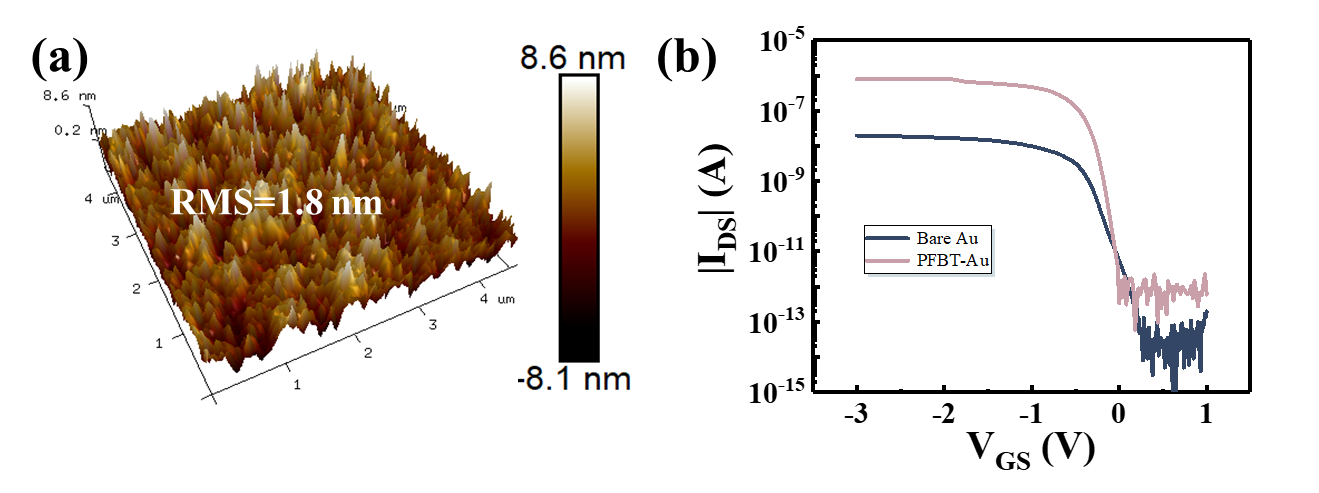


**Fig. S2** (a) The root-mean-square (RMS) roughness of the C8-BTBT film on OFETs is 1.8 nm. (b) The comparison of the transfer characteristic of the OFET with and without treated by PFBT before source and drain electrodes was deposited.

The RMS roughness of the C8-BTBT film on the OFET can be measured by atomic force microscope, shown in Fig. S2 (a). In order to reduce the conduct resistance (Rc) of the BGBC structure OFET, the surface of the source/drain (S/D) electrodes were treated by PFBT solution. The OFETs were immersed into a 5$\times$10^-3^mol$\cdot$L^-1^ solution of perfluorobenzenethiol (PFBT) self-assembled monolayers (SAMs) in ethanol for 15 min and then rinsed with ethanol ^[1]^. Compared with the transfer characteristics of the PFBT treated transistor, the Ion of the bare Au electrode transistor is smaller, and the subthreshold swing is larger, demonstrating that the PFBT SAMs effectively improves the R_c_.

**S3 Transfer characteristics of the OFETs after annealing at different hours**.


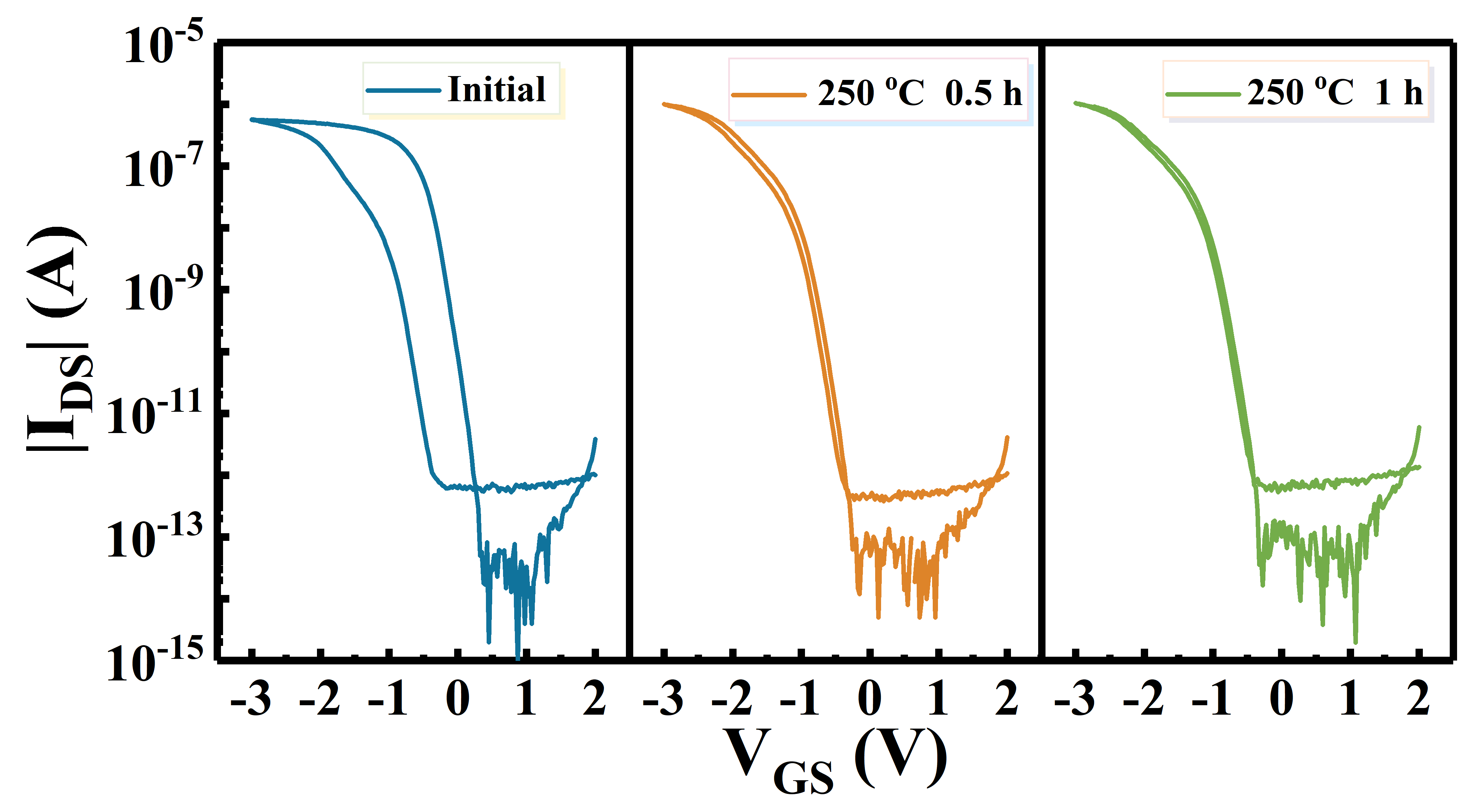


**Fig. S3** The changes in transfer characteristics of transistors after annealing for different hours in an oxygen atmosphere.

The surface of the Al_2_O_3_ dielectric deposited by ALD has various oxygen vacancies. Although the OFETs have treated by PFBT before depositing the channel to improve its contact resistance, the oxygen vacancies on the interface of the dielectric and channel can trap charges, resulting in a significant hysteresis in the transistor's transfer characteristics. As shown in Fig. S3, the transfer characteristics of the transistors after annealing in oxygen atmosphere at 250°C for 0.5 hours and 1 hour are compared with the initial characteristics before annealing. Oxygen atmosphere will fill the oxygen vacancies on the interface of Al_2_O_3_ dielectric and C8-BTBT channel at high temperatures, thereby improving the hysteresis of transistor transfer characteristics ^[2]^.

**S4** **The “box-and-whisker plot of V_th_ for the 35 different OFETs and the transfer characteristics to estimate the V_th_ of the OFETs.**


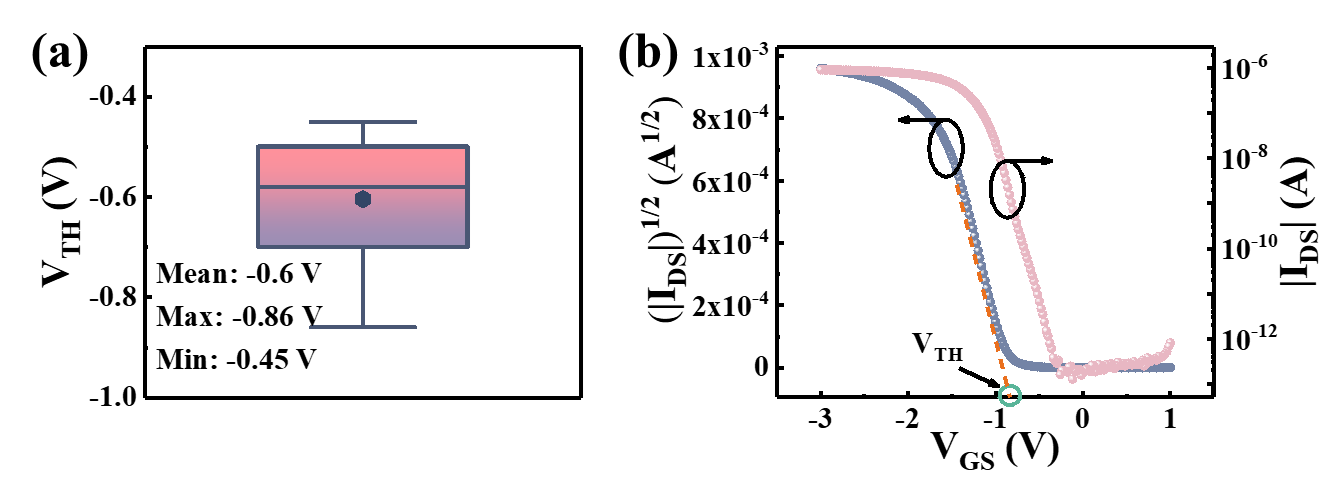


**Fig. S4** (a) The “box-and-whisker plot of V_th_ for the 35 different OFETs. (b) The transfer characteristics for the OFETs to estimate the V_th_ of the OFETs.

It can be seen from the “box-and-whisker plot of V_th_ for the 35 different OFETs, the maximum, minimum and mean V_th_ is -0.86 V, -0.45 V and -0.6 V, respectively. V_th_ can be extracted using the linear extrapolation fitting method shown in Fig. S4 (b). The V_th_ of a transistor can be obtained by drawing the intersection point of the tangent line of the relationship between the$\sqrt{|I_{DS}|}$and V_GS_ .

**S5 Histogram of subthreshold swing from 35 C8-BTBT OFETs**


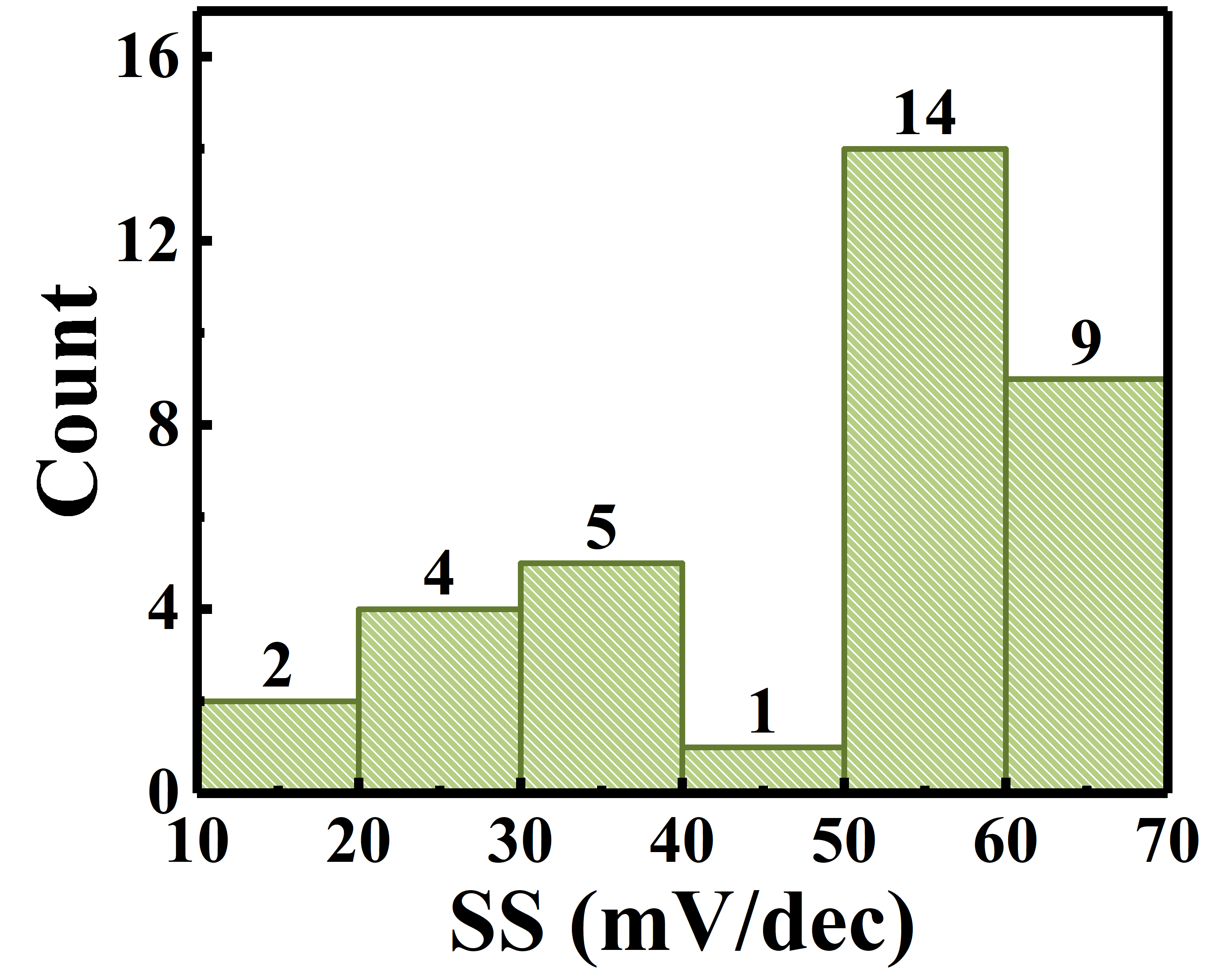


**Fig. S5** Histogram of subthreshold swing from 35 C8-BTBT OFETs, most of the OFETs can achieve the SS lower than 60 mV/dec.

The subthreshold swing shown in Fig. 2 (c) of the manuscript is calculated by the eq (1) for the subthreshold swing (SS) at a certain drain current in the transistor transfer characteristic subthreshold region ^[3, 4]^.

$SS=\frac{\partial V_{GS}}{\partial{log}_{10}I_{DS}}$ (1)

The minimum SS of the OFETs we fabricated is 17 mV/dec, and the SS of most OFETs is below 60 mV/dec. The average of the SS for the 35 OFETs is 50.9 mV/dec, lower than 60 mV/dec.

**S6 The leakage current of the OFETs**


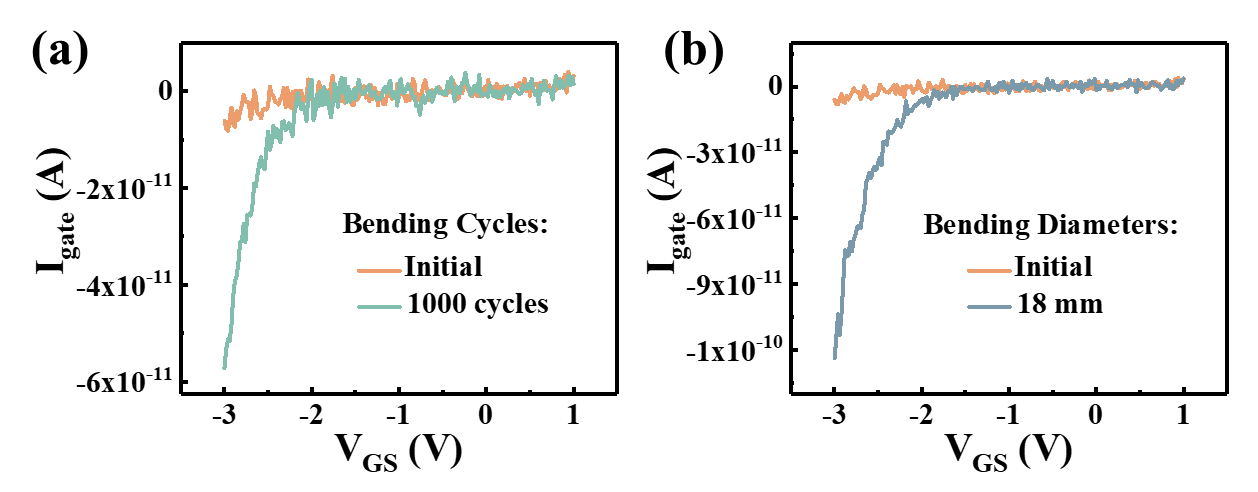


**Fig. S7** (a) and (b) are the leakage current of the OFETs under different bending cycles and bending diameters.

Compared to the initial state, the leakage current of the OFET increases after multiple bending cycles and under different bending diameters. Particularly, the leakage current shows a significant increase at higher gate voltages, which may be the cause of the degeneration in the OFET's on-state current after repeated bending.

**S7** **Schematic diagram of changes in the dielectric layer of the OFETs under bending state.**


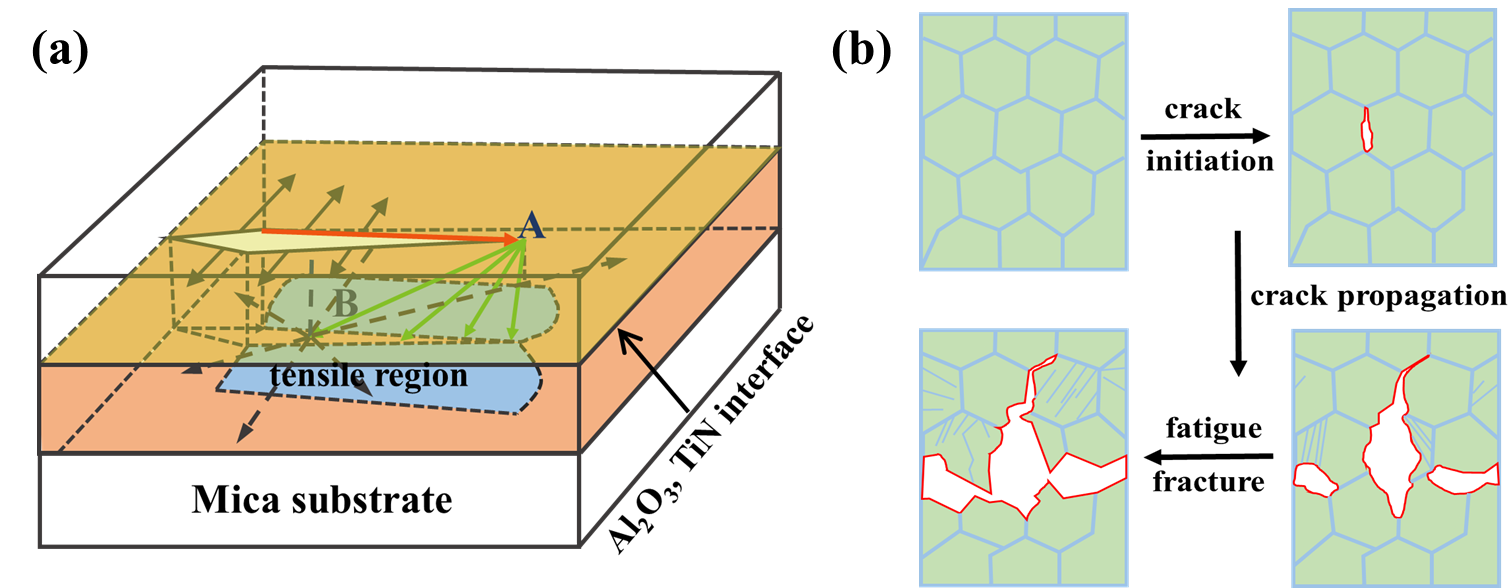


**Fig. S6** (a) Crack propagation path of the films. (b) The cracks formation along the grain boundaries of nanoparticles.

The Young's modulus of TiN is larger than that of Al_2_O_3_, degradation may occur during the repeated bending process. The stress concentration at the crack tip A may accelerate the crack propagation towards the TiN, generating the tensile region B. In the expansion process of tensile region, the crack spacing continues to increase and thus causes the fatigue fracture ^[5]^. More cracks are observed near the grain boundary, which shown as Fig. S9 (b). Cracks will cause the degradation of the insulation layer, which leads to changes in the transfer characteristics of the device.

The relationship between the bending diameter and the force applied to OFETs in finite element analysis can be expressed by the following formulas.

The single *t, E* and *ν* indicate the thickness, Young’s modulus and Poisson’s ratio, respectively. The subscripts “ƒ”and “*s”* denote the Al_2_O_3_ layer and TiN, respectively. The *ε* is the specimen strain given as eq (2) and the bending radius r given as qe (3) ^[6, 7]^:

$$\varepsilon=\left( \frac{t_{f} +t_{s}}{\text{2r}} \right)\frac{\left( \text{1+2}\eta+\chi\eta^{2} \right)}{\left( \text{1+}\eta\right)\left( \text{1+}\chi\eta\right)} (2)$$

$$r=\frac{L_{0}}{2\pi\times\sqrt{(\triangle L/L_{0} )-(\pi^{2}(t_{f} +t_{s} )^{2}/12L_{0}^{2} )}} (3)$$

Where η=t_f_/t_s_, χ=E_f_/E_s_, *r* is the flexural radius, $L_{0}$ is the length of film, which has been shown in Fig. 2 (g), and *ΔL* is the variation of the film length.

**S8 The finite element analysis model for the OFETs**


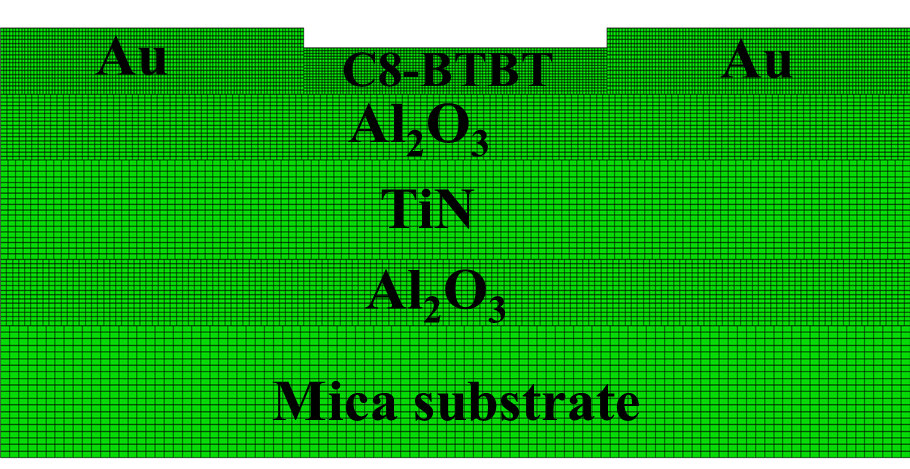


**Fig. S8** finite element analysis model for the OFETs

In order to investigate the effects of stress on crack formation, finite element analyses are conducted using ABAQUS software. A two-dimensional model is constructed, taking into account the stacked film structure (refer to Fig. S8). The model presumes ideal bonding at the interface. The mesh of the two-dimensional model of the transistor shown in Fig. S8. When conducting finite element analysis, it is necessary to densely partition the grid at locations that require high accuracy, and sparsely partition the grid in locations where high analysis accuracy is not required. In addition, grid density will be increased in locations with irregular boundary shapes to ensure smooth simulation.

**S9 Stress as a function of bending diameter at different paths.**


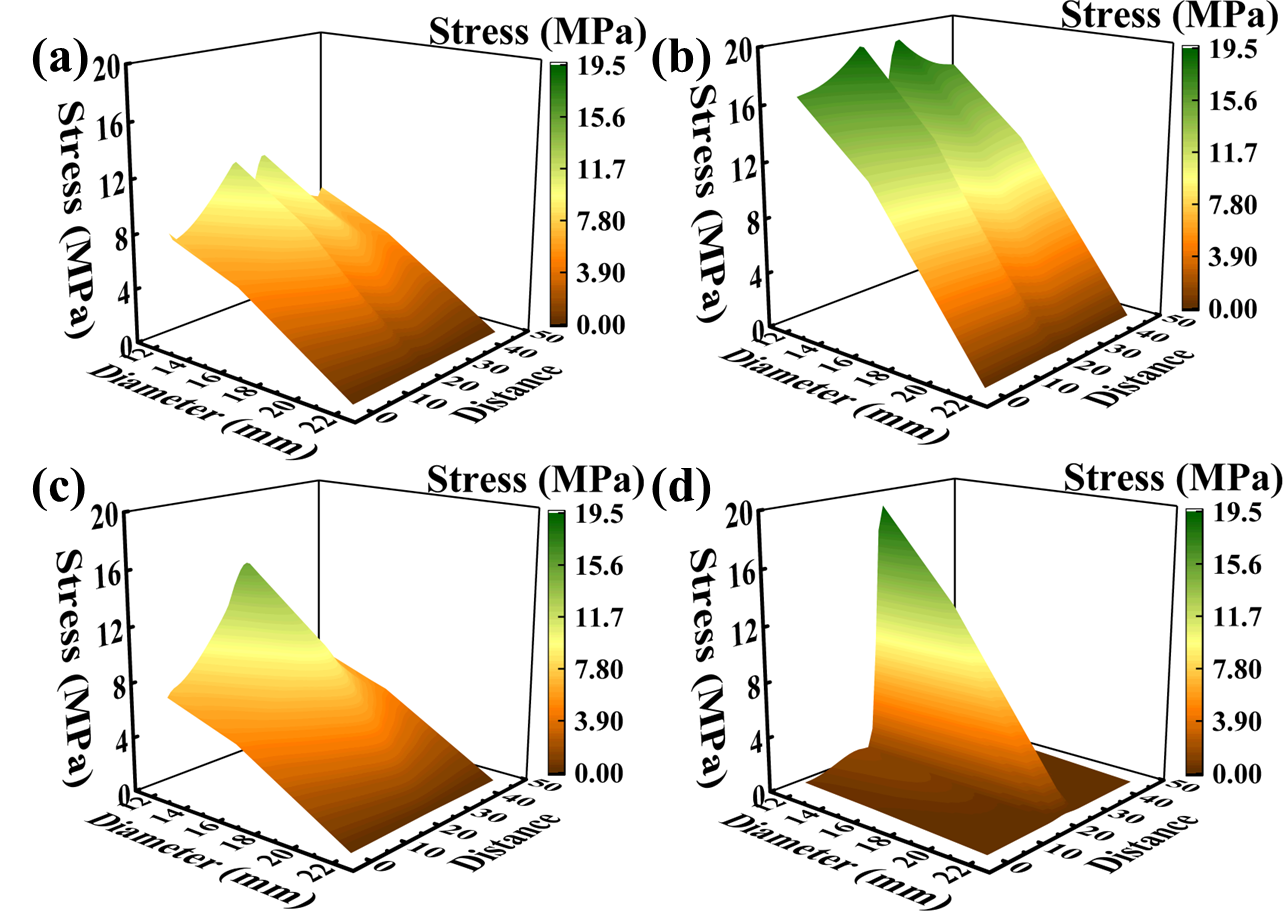


**Fig. S9** Stress as a function of bending diameter at the (a) path 1, (b) path 2, (c) path 3, (d) path 4.

As shown in Fig. S6 (a) and (b), the stress along paths 1 and 2 is relatively lower at the center of the transistor when the bending diameter is 12 mm. This is because the stress at the center is relieved after the crack formation, with the stress redistributing towards the sides. In Fig. S6 (c) and (d), for paths 3 and 4, when the bending diameter is small, the stress is primarily concentrated at the center, with a particular focus along path 4. This is attributed to the fact that during the bending simulation, the center of the bottom of the transistor is fixed, indicating that the formation of cracks relieves the stress at the Al_2_O_3_-TiN-Al_2_O_3_ interfaces, allowing the stress to propagate downward.

**S10 (a) The optical image of the 2T0C DRAM cell. (b) The variation of the I_Read_ during the endurance test process**


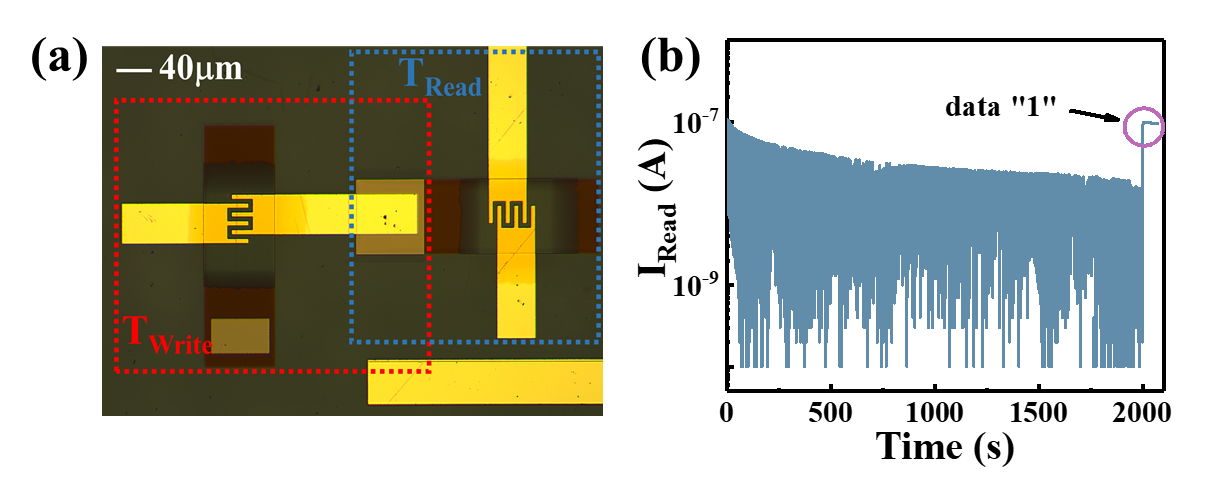


**Fig. S10** (a) The optical image of the flexible 2T0C DRAM cell constructed by the high performance OFETs. The cannel length of the flexible OFETs is 5μm. (b) The variation of the I_Read_ during 10^6^ cycles endurance test.

The optical image of the 2T0C DRAM cell structure is shown in Fig. S7 (a), and the read and write transistors that construct the 2T0C DRAM cell are all high-performance flexible OFETs. During the endurance test, the output current is read every 60 ms, and after 10^6^ cycles, data “1” was write in the 2T0C DRAM cell and the variation of the I_Read_ is shown in Fig. S7 (b).

**S11** **(a) The data retention of the 2T0C DRAM cell. (b) Real-time test photos of the 2T0C DRAM cell**


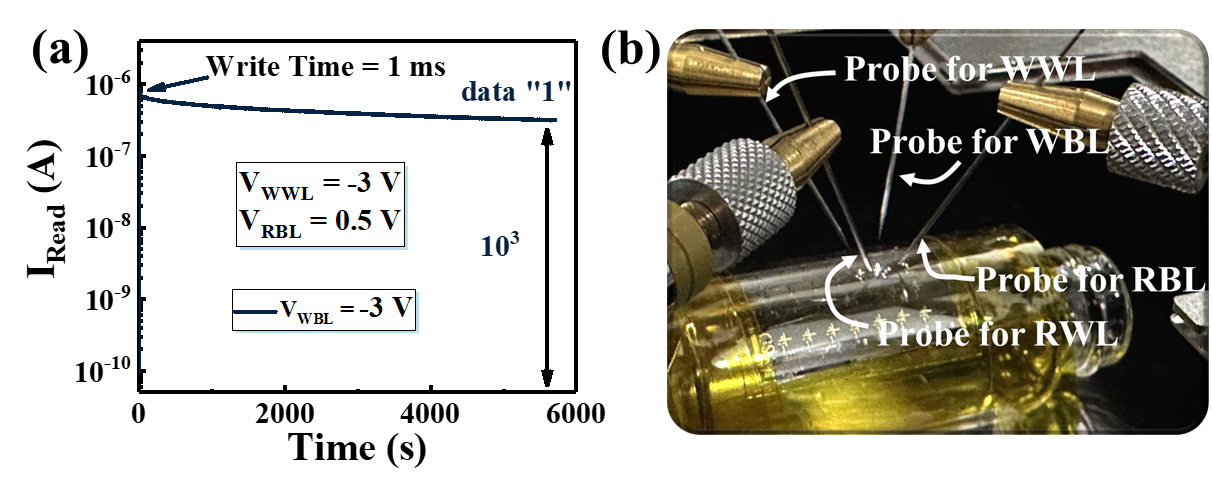


**Fig. S11** (a) The data storage characteristic of the 2T0C DRAM cell and the retention time over 6000 s. (b) Real-time test photos of the 2T0C DRAM cell.

After writing data "1," the 2T0C DRAM cell is able to retain the data for over 6000 s, with the current ratio between data "1" and data "0" exceeding 10³, demonstrating the excellent retention characteristics of our 2T0C DRAM cell. During the flexible testing of the 2T0C DRAM cell, it was bound to a small vial with a fixed diameter using adhesive tape, and signal detection was monitored using a four-probe setup.

**Table 1** Performance comparison of our 2T0C DRAM cells and recent studies

|  | Ref ^[8]^ | Ref ^[9]^ | Ref ^[10]^ | Ref ^[11]^ | Ref ^[12]^ | This work |
| --- | --- | --- | --- | --- | --- | --- |
| Channel | CNT and  IGZO | IGZO | IWO | IGZO | IWO | C8-BTBT |
| Retention (s) | 170 | 75 | 400 | 300 | 580 | 350 |
| Multilevel | No | No | No | No | No | Yes |
| Retention  standard | $\Delta$V_SN_ = 0.1V | $\Delta$V_SN_ = 0.1V | 80% drop of V_SN_ | $\Delta$V_SN_ = 0.1V | $\Delta$V_SN_ = 0.1V | $\Delta$V_SN_ = 0.1V |
| Mechanical flexibility | No | No | No | No | No | Yes |

Compared to recent researches, the 2T0C DRAM cells achieve a retention comparable to that of oxide semiconductor-based 2T0C DRAM cells under the same retention standards, while also offering multilevel storage characteristic, thereby enabling more complex data storage solutions. Besides, the 2T0C DRAM cells are constructed from OFETs and fabricated on flexible substrates, providing excellent mechanical flexibility.

**Reference**

[1] J. Zhao, W. Tang, Q. Li, W. Liu, X. Guo, *IEEE Electron Device Letters* **2017**, *38* (10), 1465, <https://doi.org/10.1109/led.2017.2742952>.

[2] Q. Liu, F. Wang, H. Lin, Y. Xie, N. Tong, J. Lin, X. Zhang, Z. Zhang, X. Wang, *Catalysis Science & Technology* **2018**, *8* (17), 4399, <https://doi.org/10.1039/c8cy00994e>.

[3] Y. Zhai, Z. Feng, Y. Zhou, S. T. Han, *Mater Horiz* **2021**, *8* (6), 1601, <https://doi.org/10.1039/d0mh02029j>.

[4] C. Jiang, H. W. Choi, X. Cheng, H. Ma, D. Hasko, A. J. S. Nathan, *Science* **2019**, *363* (6428), 719.

[5] Z.-H. Li, J.-C. Li, H.-P. J. J. o. A. Cui, Compounds, *Journal of Alloys and Compounds* **2021**, *858*, 158091.

[6] H. R. Choi, S. K. Eswaran, S. M. Lee, Y. S. J. A. A. M. Cho, Interfaces, *ACS Appl. Mater. Inter* **2015**, *7* (32), 17569.

[7] B. C. Mohanty, H. R. Choi, Y. M. Choi, Y. S. J. J. o. P. D. A. P. Cho, *J. Phys. D: Appl. Phys* **2010**, *44* (2), 025401.

[8] M. Shi, Y. Su, J. Tang, Y. Li, Y. Du, R. An, J. Li, Y. Li, J. Yao, R. Hu, presented at *2023 International Electron Devices Meeting (IEDM)*, **2023**.

[9] C. Chen, J. Xiang, X. Duan, C. Lu, J. Niu, K. Zhang, Y. Liu, N. Lu, Z. Jiao, Y. Shen, Q. Luan, G. Wang, C. Zhao, G. Yang, D. Geng, L. Li, M. Liu, presented at *2023 International Electron Devices Meeting (IEDM)*, **2023**.

[10] H. Ye, J. Gomez, W. Chakraborty, S. Spetalnick, S. Dutta, K. Ni, A. Raychowdhury, S. Datta, presented at *2020 IEEE International Electron Devices Meeting (IEDM)*, **2020**.

[11] X. Duan, K. Huang, J. Feng, J. Niu, H. Qin, S. Yin, G. Jiao, D. Leonelli, X. Zhao, Z. Wang, W. Jing, Z. Wang, Y. Wu, J. Xu, Q. Chen, X. Chuai, C. Lu, W. Wang, G. Yang, D. Geng, L. Li, M. Liu, *IEEE Transactions on Electron Devices* **2022**, *69* (4), 2196, <https://doi.org/10.1109/ted.2022.3154693>.

[12] Z. Zhao, J. Gomez, H. Ye, M. Imani, X. Yin, S. Deng, B. Melanson, J. Zhang, X. Gong, A. Abusleme, S. Datta, K. Ni, presented at *2021 IEEE International Electron Devices Meeting (IEDM)*, **2021**.
